# Combination of Synthetic Long Peptides and XCL1 Fusion Proteins Results in Superior Tumor Control

**DOI:** 10.3389/fimmu.2019.00294

**Published:** 2019-02-26

**Authors:** Natalia K. Botelho, Benjamin O. Tschumi, Jeffrey A. Hubbell, Melody A. Swartz, Alena Donda, Pedro Romero

**Affiliations:** ^1^Department of Fundamental Oncology, Faculty of Biology and Medicine, University of Lausanne, Epalinges, Switzerland; ^2^Institute for Molecular Engineering, University of Chicago, Chicago, IL, United States; ^3^Ben May Department of Cancer Research, University of Chicago, Chicago, IL, United States

**Keywords:** therapeutic cancer vaccine, antigen cross-presentation, Xcr1^+^ DC, Xcl1, synthetic long peptides

## Abstract

Cross-presenting Xcr1^+^CD8α DCs are attractive APCs to target for therapeutic cancer vaccines, as they are able to take up and process antigen from dying tumor cells for their MHCI-restricted presentation to CD8 T cells. To this aim, we developed fusion proteins made of the Xcr1 ligand Xcl1 fused to an OVA synthetic long peptide (SLP) and IgG1 Fc fragment. We demonstrated the specific binding and uptake of the Xcl1 fusion proteins by Xcr1^+^ DCs. Most importantly, their potent adjuvant effect on the H-2Kb/OVA specific T cell response was associated with a sustained tumor control even against the poorly immunogenic B16-OVA melanoma tumor. The increased tumor protection correlated with higher tumor infiltration of antigen-specific CD8+ T cells, increased IFNγ production and degranulation potential. Altogether, these results demonstrate that therapeutic cancer vaccines may be greatly improved by the combination of SLP antigen and Xcl1 fusion proteins.

## Introduction

One of the key requirements for successful therapeutic cancer vaccinations relies on the ability to target antigen to cross-presenting dendritic cells (DCs), a subtype of DCs which have the capacity to shunt a proportion of internalized antigens from the endosomal compartments to the cytosol, where they are processed for loading onto MHC class I molecules, resulting in efficient CD8^+^ T cell responses (1). The chemokine receptor Xcr1 was shown to be the main marker characterizing murine (2) as well as human cross-presenting DCs (3–5), and their superior cross-presentation capacities of soluble and cell-associated antigens has been demonstrated in both mice (2, 6, 7) and humans (3, 8). The Xcr1 chemokine receptor is co-expressed with CLEC9A (DNGR1) and the ontogeny of Xcr1-positive DCs is strictly dependent on the transcription factor Batf3 (2, 9). In mice, Xcr1 is expressed in ~80% of lymphoid organ-resident CD8α^+^ DCs as well as in ~80% of migratory dermal CD103^+^ DCs (6). In humans, XCR1 is expressed in the majority of CD141^+^ CD11c^+^ blood DCs (3) and CD141^hi^ tissue-residents DCs in dermis, liver, and lung (4, 5). Of note, Xcr1 is co-expressed with DEC205 and CADM1 (5), which suggests the strong functional role of Xcr1^+^ DCs in the cross-presentation of antigens derived from necrotic cells (10). Xcr1-expressing DCs migrate toward the chemokine Xcl1 secreted by activated CTLs, NK and NKT cells involved in the cytotoxic response (3, 11). In contrast to many chemokine ligands that bind to several receptors, Xcl1 binds exclusively to the Xcr1 receptor and is often co-secreted with Th1 profile cytokines, such as IFNγ, MIP-1α, MIP-1β, and RANTES by activated murine NK cells, Th1 cells, and CD8^+^ T lymphocytes (12).

Vaccinations involving synthetic long peptides (SLPs) have given successful results in clinical studies with cancer patients (13, 14), and are thought to avoid immunological tolerance induced by exact length MHC class I-restricted peptides. Indeed, unlike short synthetic peptides (SSP), SLPs require cellular processing and cross-presentation, which avoids suboptimal presentation by non-professional antigen presenting cells and hence efficiently induce specific CTL responses (15, 16). SLPs are generally 20–30 amino acids long and may harbor both MHC class I and class II-restricted epitopes, resulting in enhanced CTL expansion by triggering concomitant T helper responses. In addition, antigens in the form of SLPs have been compared against whole protein antigens in DC cross-presentation studies and have been shown to be better processed resulting in improved cross-priming of CD8^+^ T cell responses (17). Indeed, while whole protein traffics only to endosomal compartments which primarily promotes the priming of CD4^+^ T lymphocytes, SLPs traffic not only to endosomes, but also to cytosol, allowing the priming of both CD4^+^ and CD8^+^ T cell responses (18).

Antitumor immunity relies greatly on antigen cross-presentation to allow debris from a dying tumor cell to be processed and presented to CTLs. Nevertheless, cross-presenting DCs are present at very low frequencies in human tissues, and specific DC targeting strategies represent an important step in optimizing cancer vaccines. Strategies recently used for targeting antigen to DCs have included recombinant proteins resulting from the genetic fusion of the antigen to mAbs that target DC markers, such as DEC-205 (19) and CLEC9A (20–22), or to chemokines (23).

In this context, we aimed to target to Xcr1+ DCs tumor antigens in the form of SLP genetically fused or not to the Xcl1 chemokine. In therapeutic tumor vaccination settings, vaccination with the OVA SLP fused or not to Xcl1-Fc fusion proteins enhanced CD8^+^ T cell responses and delayed B16.OVA tumor growth. These results correlated with higher tumor infiltration of antigen-specific CTLs as well as their increased IFNγ production. These results demonstrate that therapeutic cancer vaccines may be greatly improved by Xcl1-antigen fusion proteins.

## Materials and Methods

### Mice

Age and gender-matched C57BL/6 mice were purchased from Envigo Laboratories (France). Batf3 knock out (KO) mice were bred in our facilities under specific pathogen-free conditions. All animal experimentation was performed according to ethical approval from the Canton de Vaud authorities, Switzerland. Veterinary authorization number VD2273.

### Production of Xcl1-SLP muIgG1 Fc Fusion Proteins

DNA sequences were inserted into the expression vector pMP-PB (Excellgene) by In-Fusion technique (Clontech). DNA sequences are shown in Supplementary Figure 1. Positive clones were verified by DNA sequencing (Microsynth). Middle scale protein production was performed in Chinese Hamster Ovary (CHO) cells at the Laboratory of Cellular Biotechnology of EPFL, Lausanne, Switzerland. Xcl1 fusion proteins were purified from the supernatants of 7-day CHO cultures. Purification was performed by affinity chromatography using Protein A resin (GE Healthcare, cat no 17-1281-02). Proteins were eluted with Glycine 0.1 M pH 3.0 and dialyzed against PBS overnight. After confirming their size and purity by SDS-PAGE, recombinant proteins were passed through a Mustang Q membrane (PALL Corporation) for endotoxin removal. Commercial Xcl1 was purchased from Hölzel Diagnostika Handels GmbH, Germany (item n°50677-M08B).

### *In vitro* Binding of Fusion Proteins to DCs

Spleens from naïve WT (C57BL/6) and Batf3^−/−^ mice were enriched for CD11c^+^ cells using CD11c (N418) microbeads (cat number 130-052-001, Miltenyi Biotec). DC-enriched suspensions from spleens of WT or Batf3^−/−^ mice were incubated with purified Xcl1-(OVA SLP)-Fc and Xcl1-Fc fusion proteins at 37°C for 35 min. Cells were washed and binding of fusion proteins was assessed using PE-conjugated anti-mouse IgG1 antibody.

### Chemotaxis Assay

Spleens from naïve WT (C57BL/6) mice were enriched for CD11c^+^ cells using CD11c (N418) microbeads (cat number 130-052-001, Miltenyi Biotec). 1 x 10^6^ cells (CD11c^+^ DC purity of ~50%) were resuspended in 0.1 mL of chemotaxis medium (RPMI1640, 1% BSA, 50 μM ß-ME, 100 μg/mL penicillin/streptomycin) and added to the upper chamber of a 24-transwell plate (with 8 μm pore, Corning). In the lower chamber, 0.5 mL of chemotaxis medium was added, containing either 250 ng/mL of commercial Xcl1, or 1,000 ng/mL of Xcl1-(OVA SLP)-Fc or Xcl1-Fc fusion protein to have an equimolar concentration of Xcl1 of 25 nM. After incubation for 2 h at 37°C (5% CO_2_), bottom chambers were flushed with ice-cold PBS containing 10 mM EDTA and DCs were analyzed by FACS. Cells were incubated for 5 min on ice with 2.4 G2 to block Fc receptors, Xcr1^+^ DCs were detected via incubation with Xcl1-Fc protein (19 nM) for 30 min at 37°C, followed by washing and staining with PE-conjugated anti-mouse IgG1 on ice for 30 min. Afterwards, surface markers antibodies were added in a mix, on ice, for 30 min. DCs were identified by first excluding CD3^+^ B220^+^ and CD11b^+^ cells and gating on CD11c^+^ CD8α^+^ cells.

### *In vivo* Uptake of Alexa-488-Labeled Xcl1 Fusion Proteins

Alexa-488 dye (DY-490-NHS-Ester, from Dyomics, product number 490-01) was resuspended in DMSO (the molar ratio between 1 mg of dye and 1 mg of the Xcl1 fusion proteins is 40.2, hence 40.2 μL of DMSO were added). The dye and the 10x reaction buffer (1 M Na Phosphate, 1.5 M NaCl, pH 7.1) were added to the fusion proteins at a volume ratio of 1:10, and mix was incubated at room temperature for 1.5 h in rotation and protected from light. Desalting columns (Zeba Spin desalting column, Thermo Scientific, product number 89,890) were washed with PBS by spinning 1,000 g for 2 min. The labeled proteins were added to the column and spun down. This step was repeated with the flow-through and final fusion proteins concentrations were measured by BCA.

WT and Batf3 KO mice were injected intradermally in the footpad with a mix of 50 μg of CpG and 6 μg of Alexa 488-labeled Xcl1-(OVA SLP)-Fc or Xcl1-Fc fusion proteins. Inguinal LNs were harvested 16 h post injection for measurement of uptake in different cell populations.

### Peptide Solubilization

OVA SLP was solubilized with 10% sterile DMSO and 90% sterile PBS. The OVA SLP amino acid sequence is K*ISQAVHAAHAEINEAG*RE**SIINFEKL**TEWT, which includes the MHC class I-restricted epitope (in bold) and the MHC class II-restricted epitope (in italic).

### Immunizations

Vaccine formulations were prepared sterile, immediately before injections. Mice were immunized with a volume of 30 μL intradermally in the hind paw, on the ipsilateral side of the tumors.

### Tumor Engraftment

Mice were engrafted subcutaneously in the left flank either with 1 x 10^6^ EG7 or 2 x 10^5^ B16.OVA cells, or 1 x 10^5^ B16.WT. Tumor volumes were monitored every 2 days and were calculated using the following formula: (length × width × thickness)/2.

Tumor Digestion: Tumors were harvested and digested using the tumor dissociation kit from Miltenyi Biotec (cat number 130-096-730), according to manufacturer's instructions. Cells were then stained for flow cytometry.

### Intradermal Vaccination

Mice received equimolar amounts of Xcl1 and OVA SLP antigen injected intra-dermally in the footpad. Doses were the following: 20 μg of Xcl1-(OVA SLP)-Fc; or 17.6 μg of Xcl1-Fc + 1.3 μg of free OVA SLP; or 1.3 μg of free OVA SLP + 5.9 μg free Xcl1; or 1.3 μg of free OVA SLP. All mice received 50 μg CpG-B (ODN 1826, U133-L01A; Trilink Biotechnologies).

### Isolation of TILs

Tumors were digested as described above. Samples were then diluted in 7 mL of complete DMEM and added to 5 mL of Lymphoprep (cat number 1114547, Axis-Shield), followed by a centrifugation of 1,800 rpm for 20 min. Cells at the interphase were collected, washed once, and plated in a 96-well plate for *in vitro* peptide restimulation.

*In vitro* peptide restimulation and Intracellular Cytokine Staining: TILs were incubated at 37°C for 1 h with 10 μM SIINFEKL and anti-mouse CD107a (LAMP1) antibody-FITC was also added (1/100) to wells. After 1 h, 1 μg/mL GolgiPlug and GolgiStop (BD biosciences) were added to the wells and TILs were then incubated for a further 4 h at 37°C before intracellular cytokine staining. Cells were permeabilized and stained using the Cytofix/Cytoperm kit (BD Biosciences), according to manufacturer's instructions and stained for intracellular IFNγ and TNFα.

Calculation of the CD8/Tregs ratio: TILs were counted under the microscope before surface/intracellular staining and FACS acquisition. CD8/Treg ratio were calculated using the FACS percentages of tetramer^+^ CTLs and CD4^+^ CD25^+^ FoxP3^+^, and total TIL numbers.

### Flow Cytometry

Blood and spleen samples were treated with Red Blood Cell Lysis Solution (Qiagen) for 15 min at 37°C and 3 min at room temperature, respectively, before staining. LIVE/DEAD Aqua fluorescent stain (Invitrogen) was used to discriminate between live and dead cells. For tetramer staining, samples were incubated with phycoerythrin (PE)-conjugated SIINFEKL-H-2k^b^ multimers (TC Metrix, Switzerland) for 35 min at room temperature. Samples were washed and incubated on ice for 30 min with CD8α-PerCp Cy5.5 (clone 53.6.7–eBioscience), CD3–PE Cy7 (clone 145.2C11–eBioscience), CD4–FITC (clone GK1.5–produced in house, Ludwig Cancer Research). For *in vitro* binding and chemotaxis assays the following antibodies were used: IgG1–PE (clone A85-1–BD biosciences), B220–Pacific blue (clone RA3-6B2 - LICR), CD8a–PerCp Cy5.5 (clone 53.6.7–eBioscience), CD3–PE Cy7 (clone 145.2C11–eBioscience), CD11c–eFLuor 660 (clone N4/18–eBioscience), CD11b–Alexa700 (clone M1/70–eBioscience), CD103–PE. Data were acquired on a LSRII or LSRII (SORP) and FACS analyses were done with Flow Jo software.

### Statistical Tests

Statistical analyses were performed using GraphPad Prism 7 software (GraphPad Software, La Jolla, CA). Normally distributed data were compared using one-way ANOVA or two-way ANOVA (**Figures 3A,B, 5A**). Multiple comparisons were corrected using Tukey tests. Normality was tested with a Shapiro-Wilk test. On the graphs, data represent mean ± SE (^*^p < 0.05; ^**^p < 0.01; ^***^p < 0.001; ^****^p < 0.0001).

## Results

### Xcl1-(OVA SLP)-Fc Fusion Proteins Bind to CD11c^+^ CD8α^+^ DCs and Induce Chemotaxis of Xcr1^+^ DCs

**Figure 1:**
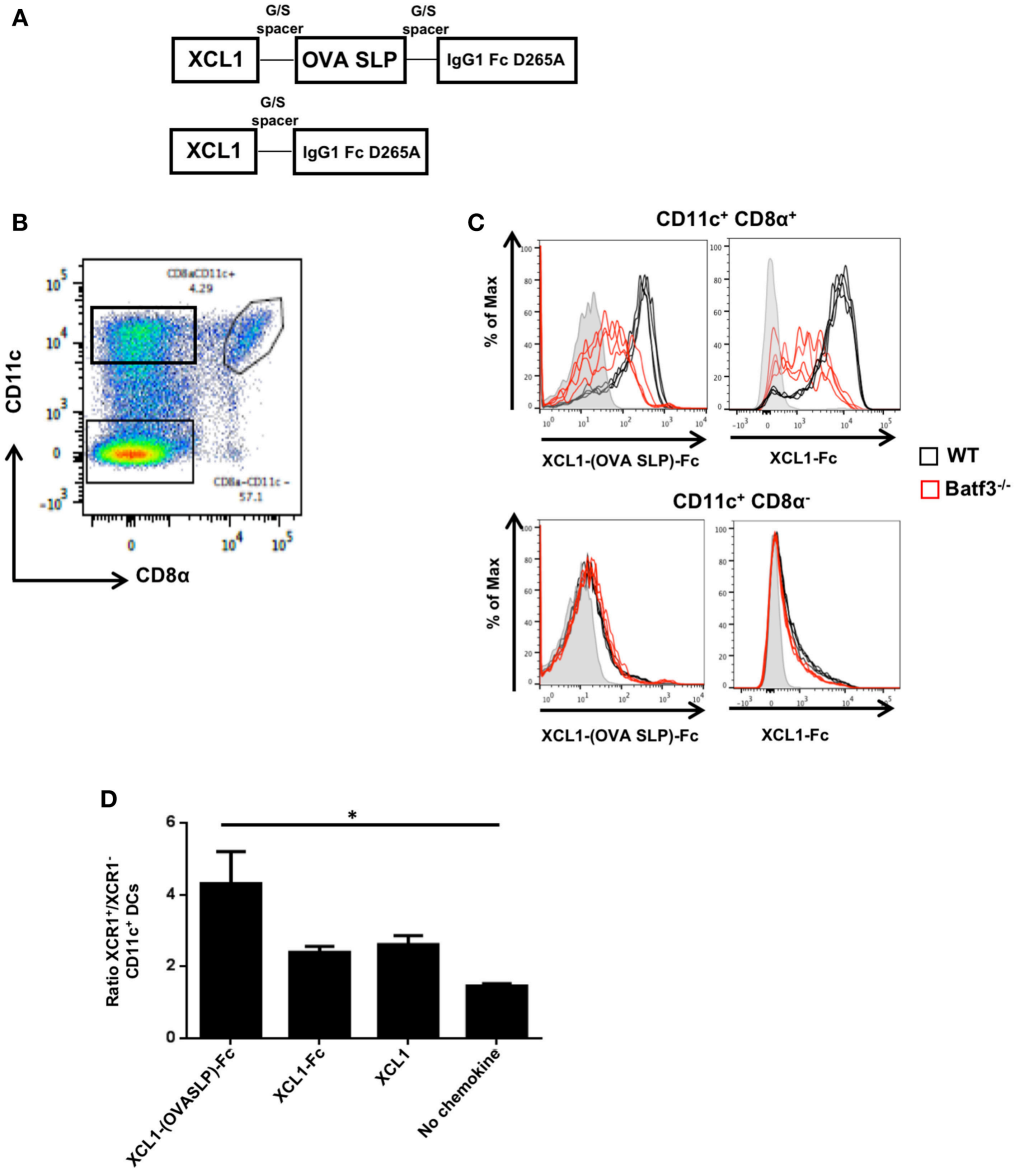
*In vitro* characterization of Xcl1-(OVA SLP)-Fc fusion proteins. **(A)** Design of Xcl1-(OVA SLP)-Fc fusion proteins. The OVA SLP was fused to the C-terminus of the murine Xcl1 amino acid sequence via an uncharged glycine/serine linker. The C-terminus of OVA SLP was connected to the murine IgG1 Fc, carrying the D265A mutation. **(B)** Gating strategy to identify CD8α DCs. **(C)** CD11c-enriched DCs from splenocytes of naive WT (C57BL/6) (black line) or Batf3^−/−^ (red line) mice were incubated with purified Xcl1-(OVA SLP)-Fc or Xcl1-Fc fusion proteins. Binding of XCL1 fusion proteins was assessed using a fluorescent anti-mouse IgG1 antibody. Gray histograms represent control wells without fusion proteins. Each line represents a replicate. Results are representative of two independent experiments. **(D)** *In vitro* chemotaxis assay performed with WT splenic DCs (enriched ~50% CD11c^+^). Migration of DCs was assessed toward Xcl1-(OVA SLP)-Fc fusion proteins at 25 nM (calculated based on the content of Xcl1 in the reagents). Results are expressed as the ratio between the number of Xcr1^+^ and Xcr1^−^ CD11c^+^ CD11b^−^ DCs, which migrated toward Xcl1 fusion proteins or commercial Xcl1. Data are shown as mean ± SEM. Results are representative of three independent experiments. ^*^p < 0.05.

With the aim to optimize synthetic long peptide (SLP) vaccines by targeting the antigen to Xcr1^+^ cross-presenting DCs, a recombinant fusion protein was produced with the ovalbumin (OVA) SLP antigen fused to the Xcl1 chemokine, followed by the murine IgG1 Fc for stability, dimerization and purification purposes (Supplementary Figure 1). We opted for an Fc part harboring the Asp to Ala mutation at amino acid position 265, which prevents its binding to Fc receptors (24). A recombinant protein lacking the OVA SLP antigen (Xcl1-Fc) was also produced to evaluate the potency of Xcl1-mediated antigen targeting (Figure 1A). The fusion proteins were tested for their capacity to bind to CD11c^+^-microbeads purified CD8α^+^ DCs from spleen (Figure 1B). CD11c^+^-enriched DCs from naïve WT and Batf3^−/−^ mice were incubated with the Xcl1-(OVA SLP)-Fc fusion proteins at 37°C, and specific binding was detected with a fluorescently-labeled anti-IgG1-Fc antibody. Significant binding of Xcl1 fusion proteins was seen in WT mice, when gating on CD11c^+^ CD8α^+^DCs, while some heterogenous non-specific binding was observed on the remaining CD8α^+^ cells from Batf3^−/−^ mice, which are deficient in Xcr1^+^ DCs (25) (Figure 1C). Similarly, the Xcl1 fusion proteins did not bind to CD8α negative WT and Batf3 KO CD11c^+^ DCs (Figure 1C), supporting the binding specificity to CD11c^+^ CD8α^+^ DCs, 80% of which express Xcr1. To test whether the Xcl1-(OVA SLP)-Fc fusion protein was capable of inducing chemotaxis of Xcr1^+^ DCs, trans-well migration experiments were performed with 1 x 10^6^ CD11c^+^ enriched DCs in the upper chamber and medium containing 25 nM of Xcl1 fusion proteins or commercial Xcl1 in the bottom well. After a 2-h incubation at 37°C, analysis of the bottom well-showed that Xcr1^+^ DCs had migrated between 2 and 4-fold more than Xcr1^−^ DCs in all wells containing Xcl1 fusion proteins or free Xcl1 (Figure 1D). Overall, these data demonstrated that the Xcl1-(OVA SLP)-Fc and Xcl1-Fc proteins induced chemotaxis to a similar extent as the native chemokine Xcl1 (Figure 1D).

### XCL1-(OVA SLP)-Fc Fusion Protein Bind *in vivo* to CD11c^+^ CD8α^+^ LN-Resident DCs

**Figure 2:**
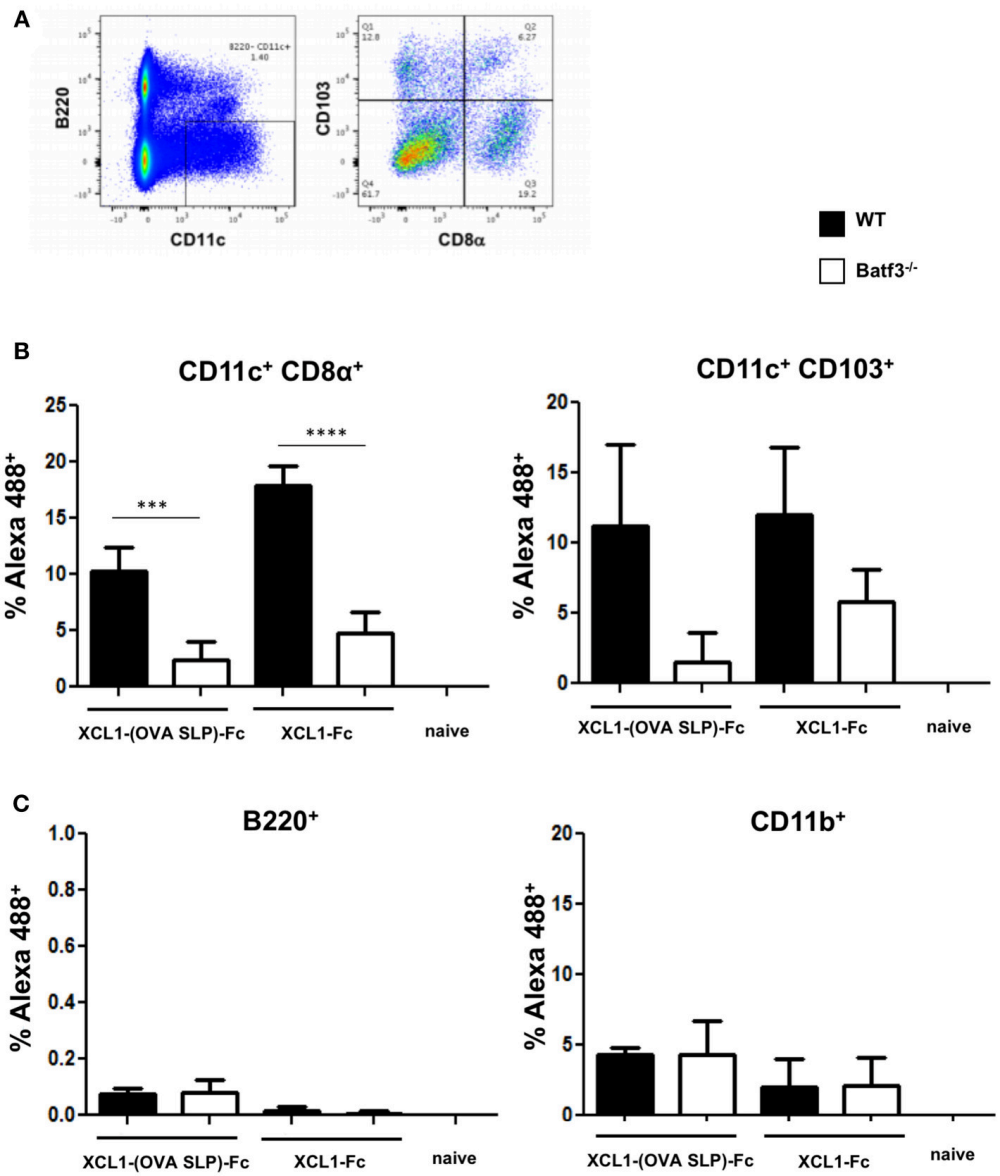
*In vivo* uptake of Xcl1-(OVA SLP)-Fc in skin draining LN. WT and Batf3 KO mice were injected intradermally in the footpad with a mix of 50 μg of CpG and 6 μg of Alexa 488-labeled Xcl1-(OVA SLP)-Fc or Xcl1-Fc fusion proteins. Inguinal LNs were harvested 16 h post injection and the uptake of labeled fusion proteins was measured in different populations of APCs, **(A)** gating strategy for identifying CD103+ and CD8α subtypes in CD11c^+^B220^neg^DCs isolated from inguinal LNs. **(B)** Uptake of labeled Xcl1-fusion proteins by CD8α DCs (left), CD103+ DCs (right), and **(C)** B220+ B cells (left), and CD11b macrophages (right). Data are shown as mean +/- SEM (*n* = 3–4 mice/group). Results are representative of two independent experiments. ^***^p < 0.001, ^****^p < 0.0001.

To investigate *in vivo* which population of DCs will preferentially bind the Xcl1-(OVA SLP)-Fc fusion proteins, Xcl1-Fc and Xcl1-(OVA SLP)-Fc were fluorescently-labeled with Alexa 488 and injected intradermally into WT or Batf3^−/−^ mice. Skin draining LNs were harvested 16 h post immunization and analyzed for the presence of the fusion protein in different subsets of CD11c^+^ DCs (Figure 2A). In WT mice injected with 6 μg of labeled Xcl1-(OVA SLP)-Fc, about 10% of CD8α^+^ LN-resident were Alexa 488 positive, compared to only 2% in Batf3^−/−^ mice (Figure 2B). Increased uptake of Alexa 488-labeled Xcl1-Fc by WT CD8α^+^ was also observed, as shown by 18% compared to 4.7% in the same DC population in Batf3^−/−^ mice. With regards to CD103^+^ DCs, there was a tendency for increased uptake of the fusion proteins by WT mice, although not significant due to a large dispersion. Importantly, B cells, which are negative for Xcr1 expression, did not bind the Xcl1 fusion proteins, while < 5% of phagocytic CD11b^+^ DCs, also negative for Xcr1, became Alexa 488 positive for the Xcl1 fusion proteins both in WT and Batf3^−/−^, indicating a non-specific uptake (Figure 2C). Altogether, these results suggest that the Xcl1-(OVA SLP)-Fc fusion proteins were preferentially and specifically taken up by the Xcr1^+^ expressing CD8α^+^. Representative profiles of *ex vivo* Alexa 488^+^-labeled cells are shown in Supplementary Figure 3.

### Therapeutic Vaccines Involving Xcl1 Fusion Proteins Lead to Regression of OVA-Expressing Tumors

**Figure 3:**
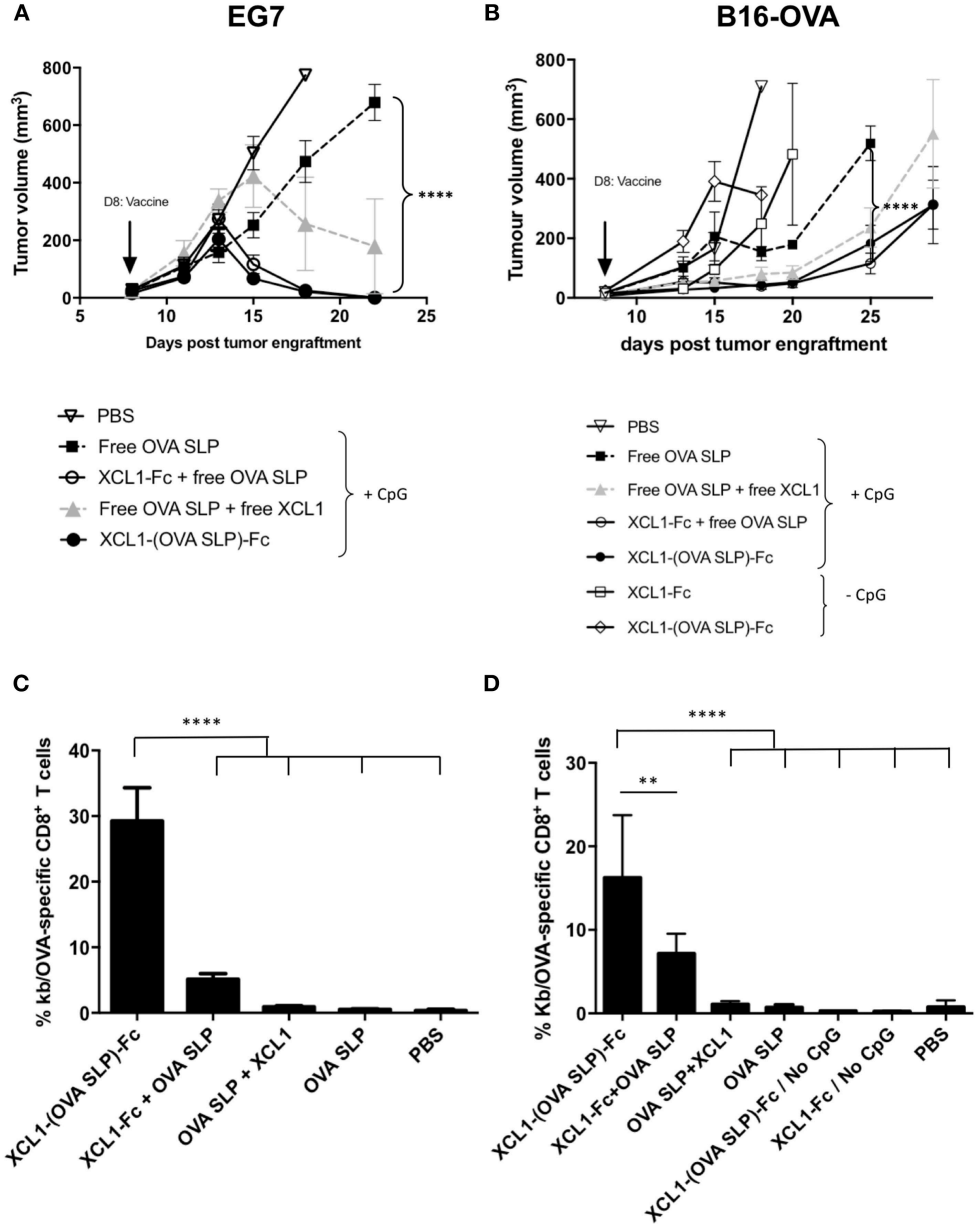
Anti-tumor immunity upon Xcl1-(OVA SLP)-Fc therapeutic vaccinations in tumor bearing mice. **(A)** Tumor growth of the T lymphoma EL4-OVA cell line (EG7) grafted s.c. on the flank of mice (1 x 10^6^ cells), followed on day 7 by the i.v. transfer of 10^5^ OT-I cells and on day 8 by i.d. vaccination on the left foot (arrow). Cohorts of mice received equimolar amounts of Xcl1 and OVA SLP antigen as described in Materials and Methods. All cohorts received 50 μg of CpG-ODN, except the PBS control **(B)** Tumor growth of B16.OVA tumors engrafted s.c. on the flank of mice (2 x 10^5^ cells), followed on day 7 by the i.v. transfer of 10^5^ OT-I cells and on day 8 by i.d. vaccination on the left foot (arrow), as described in **(A)**. Graphs represent tumor kinetic as the mean of tumor volume of 6 mice per group ± SEM. *n* = 6 (except in groups with only fusion protein where *n* = 3). Results are representative of three independent experiments. **(C)** Frequencies of H-2Kb/OVA tetramer positive CD8^+^ T cells in the blood 7 days after vaccination of EG7 and **(D)** of B16.OVA tumor bearing mice as described, respectively, in **(A,B)**. Data are shown as mean ± SEM (*n* = 6 mice/group). Results are representative of two independent experiments. ^**^p < 0.01, ^****^p < 0.0001.

Given that cancer vaccines are ultimately evaluated for their capacity to protect against tumors, the Xcl1 fusion proteins were tested in therapeutic settings against the OVA-expressing EL-4 lymphoma model (EG7). Gender and age-matched C57BL/6 mice were engrafted subcutaneously on day 0 with 1 x 10^6^ EG7 cells (Figure 3A). On day 7, when tumors were established and measurable, mice received an adoptive cell transfer of 10^5^ OT-I cells, followed on day 8 by intradermal vaccination with the Xcl1 fusion proteins or with free OVA SLP +/- Xcl1. Except for the untreated group, all mice received 50 μg of CpG-ODN. In both cohorts vaccinated with the Xcl1-(OVA SLP)-Fc fusion proteins, all tumors started to shrink 5 days post immunization. In contrast, in mice receiving free OVA SLP + free Xcl1, tumor volumes started to decrease only by day 15 but did not disappear, while in mice receiving only the OVA SLP and CpG, only a delay in tumor growth was obtained but no transient decrease of tumor volumes (Figure 3A).

In view of the potent antitumor activity of Xcl1 fusion proteins observed in the EG7 tumor model, we assessed the tumor protective immunity of the Xcl1-mediated tumor vaccine in the less immunogenic B16-OVA melanoma tumor model. Mice were grafted on day 0 with 2 x 10^5^ B16.OVA cells and on day 7, when all tumors were reaching an average volume of 30 mm^3^, mice received an adoptive cell transfer of 10^5^ naïve OT-I cells, followed on day 8 by the intradermal vaccinations as described for the EG7 challenge (Figure 3A). A significant tumor growth delay was obtained in cohorts vaccinated with Xcl1-(OVA SLP)-Fc and OVA SLP + Xcl1-Fc fusion proteins, as compared to mice not receiving Xcl1 (OVA SLP and CpG only), while only a tendency to a higher delay was observed against the OVA SLP + free Xcl1 cohort (Figure 3B). To assess a non-specific adjuvant effect of the fusion proteins due to potential traces of endotoxin, two groups were vaccinated with the Xcl1-Fc and Xcl1-(OVA SLP)-Fc fusion proteins without CpG. However, both groups of mice showed fast tumor growth (Figure 3B), confirming the adjuvant effect of Xcl1 fusion proteins. As seen in the blood on day 7 post-vaccination in both EG7 and B16.OVA tumor challenge experiments, the vaccination with Xcl1-(OVA SLP)-Fc fusion proteins plus CpG led to similar expansions of OVA-specific CTLs, which was best with Xcl1-(OVA SLP)-Fc, when compared to any other cohort, likely resulting from the co-delivery of the antigen to cross-presenting DCs via its fusion to Xcl1 (Figures 3C,D). Combined immunization with the mixture of the fusion Xcl1-Fc protein and the free OVA SLP + CpG still resulted in a significantly better CTL expansion than in the group receiving free Xcl1 mixed with the OVA SLP + CpG, which only showed a trend for higher OVA-specific CTLs as compared to only OVA SLP + CpG.

### Tumors of Mice Vaccinated With Xcl1 Fusion Proteins Show Higher Infiltration of OVA-Specific CD8^+^ T Cells Characterized by an Increased Functionality

**Figure 4:**
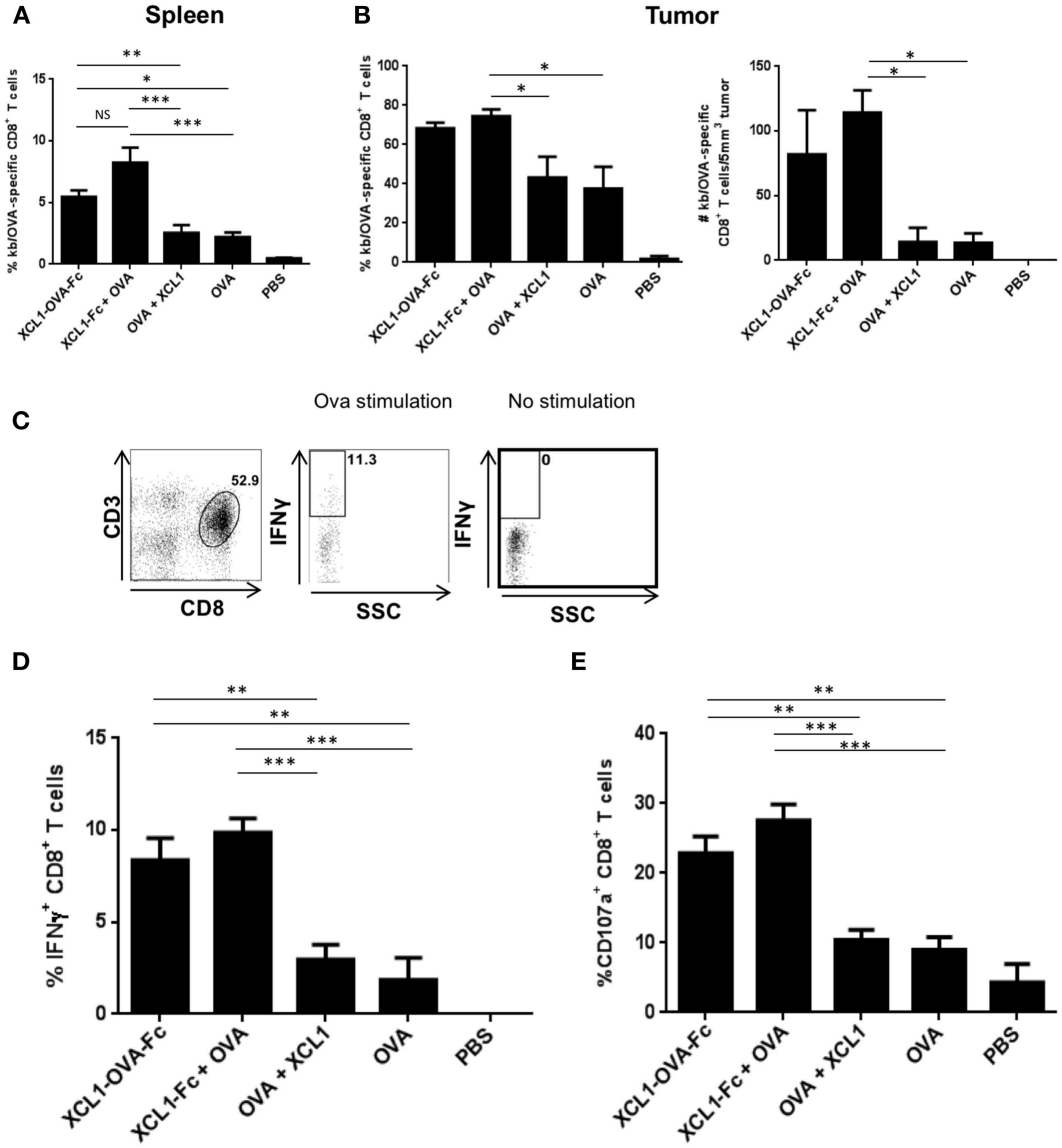
*Ex vivo* function of TILs following Xcl1-fusion proteins vaccination. **(A)** Frequencies of OVA-specific CD8^+^ T cells in the spleens harvested 10 days post vaccination of mice challenged with B16.OVA on day 0, adoptively transferred with OT-I cells on day 7 and vaccinated on day 8. Data is shown as mean ± SEM (*n* = 5 mice/group). Results are representative of two independent experiments. **(B)** Frequencies of H-2Kb/OVA tetramer positive CD8^+^ T cells present in the B16.OVA tumors of vaccinated mice (left panel), and absolute number of SIINFEKL-specific CD8^+^ T cells per 5 mm^3^ of tumor mass (right panel). Data is shown as mean ± SEM (*n* = 5 mice/group). Results are representative of two independent experiments. **(C)** Representative plots depicting the IFNγ content of CD3^+^CD8^+^ T cells isolated from B16.OVA tumors 10 days post-vaccination and restimulated with or without SIINFEKL peptide. **(D)** Frequencies of IFNγ^+^ CD8^+^ T cells and **(E)** of CD107a^+^ CD8^+^ T cells isolated from B16-OVA tumors. Data is shown as mean ± SEM (*n* = 5 mice/group). Results are representative of two independent experiments. ^*^p < 0.05, ^**^p < 0.01, ^***^p < 0.001.

In order to dissect the mechanisms by which therapeutic vaccinations using Xcl1 fusion proteins showed better tumor control, B16.OVA tumors from mice immunized as described in Figure 3B, were harvested 10 days post vaccination in order to quantify TILs and characterize their functionality. Frequencies of OVA-specific CD8^+^ T cells in the spleen (Figure 4A) and in the tumors (Figure 4B, left panel) were higher in the cohorts of mice vaccinated with Xcl1 fusion protein as compared to the other cohorts. When normalized by the tumor volume, mice vaccinated with the Xcl1 fusion proteins also showed higher numbers of OVA-specific CD8^+^ T cells, as compared to cohorts vaccinated with free OVA SLP + CpG, with or without free Xcl1 (Figure 4B right panel). Upon *in vitro* restimulation of tumor-infiltrating lymphocytes (TILs) with SIINFEKL as illustrated in Figure 4C, we found that cohorts vaccinated with Xcl1 fusion proteins showed higher frequencies of IFNγ^+^ TILs than the other cohorts (Figure 4D). Furthermore, increased frequencies of CD8^+^ TILs expressing the lysosomal marker CD107a were also observed (Figure 4E), associated with higher CD107a mean fluorescence intensity (data not shown), indicative of increased degranulation capacity. Altogether, these results suggest not only a higher frequency but also a higher functionality of CTLs within tumors of mice vaccinated with Xcl1-OVA SLP-Fc or Xcl1-Fc + free OVA SLP.

### Immunization With Xcl1 Fusion Proteins Generates an Endogenous OVA CD8^+^ T Cell Response as Efficient as Upon OT-1 T Cell Transfer

**Figure 5:**
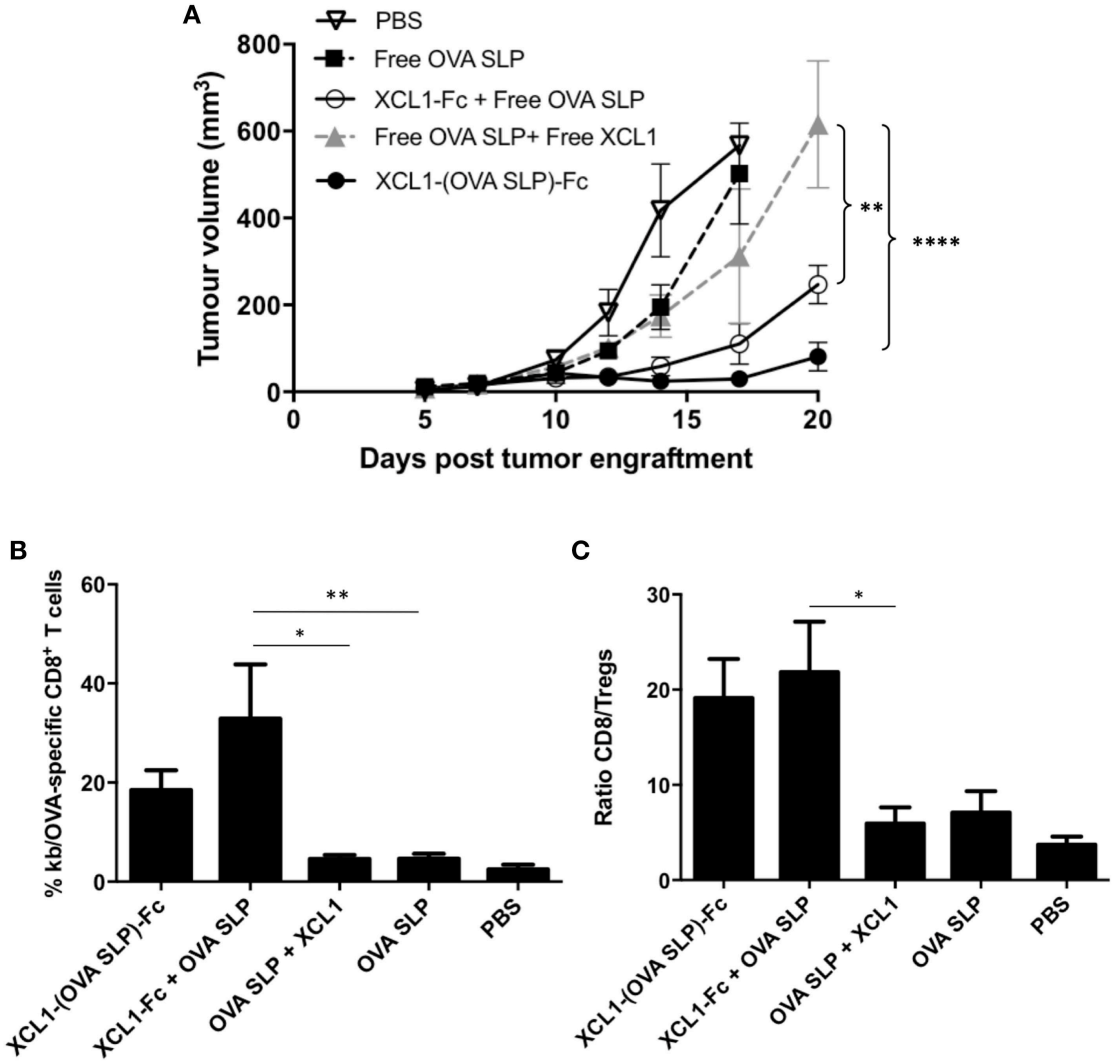
Effective therapeutic vaccinations with Xcl1-(OVA SLP) fusion proteins even in the absence of OT-1 T cell transfer. **(A)** B16.OVA tumor growth of C57BL/6 mice engrafted subcutaneously on the left flank with of 2 x 10^5^ B16.OVA cells followed by intradermal vaccination on day 3. Data is shown as mean ± SEM (*n* = 5–6 mice/group). Results are representative of three independent experiments. **(B)** Frequencies of SIINFEKL-specific CD8^+^ T cells present in the B16.OVA tumors harvested 10 days post vaccination. **(C)** Ratio of SIINFEKL-specific CD8^+^ T cells vs. Tregs within B16.OVA tumors. Data is shown as mean ± SEM (*n* = 5–6 mice/group). Results are representative of two independent experiments. ^*^p < 0.05, ^**^p < 0.01, ^****^p < 0.0001.

To be closer to a clinical situation, we wanted to assess the tumor protection capacity of the Xcl1 recombinant proteins in therapeutic vaccinations without OT-1 adoptive cell transfer. To this aim, C57BL/6 mice were grafted s.c. with 2 x 10^5^ B16.OVA melanoma cells as described in Figure 3. Mice were vaccinated 3 days later, when tumors were all visible in the flank of the mice. As in the previous experiment involving OT-1 T cell transfer, mice vaccinated with Xcl1-(OVA SLP)-Fc fusion protein showed better control of B16.OVA tumor growth, compared to other cohorts (Figure 5A). Mice were bled 7 days after vaccination and the percentages of OVA-specific CD8^+^ T cells followed the same pattern as seen upon OT-1 cell transfer, with the highest percentages in the Xcl1-(OVA SLP)-Fc and Xcl1-Fc + OVA SLP-immunized mice (Supplementary Figure 2). Strikingly, when comparing tumor growth kinetic with or without OT-1 T cell transfer (Figures 3A, 5A), the tumor control was quite similar, despite a 10-fold lower frequency of endogenous OVA-specific T cells, as seen in the blood on day 7 post vaccination (Supplementary Figure 2). Moreover, when analyzing tumors 10 days post vaccination, we observed that the frequency of OVA-specific CTLs infiltrating the tumors of Xcl1-(OVA SLP)-Fc- and Xcl1-Fc + OVA SLP-immunized mice was only 2–3 fold lower in the absence of OT-1 cell transfer (Figure 5B), which confirmed their efficient homing to the tumor, as compared to mice vaccinated with free OVA SLP + free Xcl1. In addition, these settings also revealed that the ratio between antigen-specific CD8^+^ T cells and Tregs inside the tumor mass was 4-fold higher in Xcl1 fusion proteins-vaccinated cohorts when compared to mice vaccinated with free OVA SLP with or without free Xcl1 (Figure 5C). Representative profiles of the gating strategy for identifying T regs and OVA-specific CTLs are shown in Supplementary Figure 4.

## Discussion

The goal of therapeutic cancer vaccines is to elicit a tumor-specific T cell-mediated immune response, and their success will rely on the use of adjuvants able to break immune tolerance, given that in most cases tumor antigens are derived from self-antigens. In that context, cross-presenting DCs are the APCs of choice, as they are the only subtype of DCs capable of diverting part of endocytosed antigens, such as peptides, from the endocytic pathway to the cytosolic compartment where antigen is degraded by the immunoproteasome before being loaded on to MHC class I molecules for CD8^+^ T cell presentation (1). The aim of the present study was to develop a strategy to harness these essential cross-presenting DCs.

To do so, we took advantage of the uniquely selective expression of the Xcr-1 chemokine receptor by cross-presenting DCs, essential for their chemotaxis toward primed T cells at the site of infection. We showed that fusion proteins of Xcl1, fused or not to a peptide antigen and dimerized on a Fc domain were significantly internalized by lymph node-resident CD8α^+^ DCs, and a trend for preferential uptake by migratory CD103^+^ DCs was also observed [of which ~80% express the Xcr1 chemokine receptor (7)]. With regard to T cell antigen priming, we have recently shown that the magnitude of tumor control depends on the avidity of TAA recognition by tumor-infiltrating T cells (26). In the present study, we have used the OVA antigen as a surrogate neoantigen, since it is not subjected to central tolerance and hence allows the priming and recruitment of high affinity T cells to the tumor site. Indeed, therapeutic vaccination with the Xcl1-(OVA SLP)-Fc fusion proteins was able to induce complete tumor regression in the EG7.OVA model and a delayed tumor growth in the more stringent B16.OVA melanoma model.

Previous studies have exploited Xcr1-antigen targeting either in the context of Flu (27) or cancer vaccines. For instance, Xcl1 or an anti-Xcr1 mAb have been fused to the full OVA protein and tested in antitumor vaccinations, albeit in a tumor prophylaxis setting (28). During the same year, another study has targeted Xcr1^+^CD103^+^ DCs via laser-assisted intradermal ear vaccination with Xcl1-OVA fusion protein on day 3 post tumor graft (29). We now further demonstrate the vaccine potency of Xcl1-antigen fusion proteins when injected on day 7 post-tumor graft, when EG7 tumors or the more aggressive B16.OVA tumors are fully established. Our study shows the monitoring of tumor growth over a long period of time and, instead of LPS, our vaccine formulation included the TLR9 ligand CpG-ODN, which is a clinically accepted adjuvant (30). Moreover, our study shows the extent to which vaccination impacts the immune response within B16.OVA tumors, which showed a potent recruitment of OVA-specific T cells to the tumor even in the absence of OT-1 T cell transfer. In addition to their tumor targeting, these tumor-specific CTLs also showed better effector functions, such as IFNγ production and degranulation capacity.

Various strategies have used other surface markers to deliver antigens to cross-presenting DCs, such as DEC205 (19) and CLEC9A (20). Moreover, chemokine receptors common to several subpopulations of DCs were also used to deliver antigens fused to a chemokine such as the gp100 melanoma antigen fused to CCL20 (31). The authors showed that such fusion proteins are endocytosed via binding to the chemokine receptor and are delivered to the cytosol for proteasomal processing, resulting in their loading on MHC class I molecules in a TAP-1-dependent manner, leading to potent tumor control. Alternative strategies to target antigens to other subsets of DCs have also been shown, for example by using glycoliposomes targeting DC-SIGN^+^ DCs (32), or adenylate cyclase-based vector (CyaA) that target CD11b^+^ DCs (33). Unfortunately, the large variability between all these vaccination protocols does not allow evaluating which DC marker is the most efficient for T cell priming.

In both of our tumor models, the frequencies and functionality of tumor infiltrating T cells as well as associated tumor control were similar, whether the OVA SLP was fused with the Xcl1-Fc or was co-delivered, which suggests that the signaling machinery induced by the internalization of the cargo via the Xcr1 receptor was instrumental for efficient antigen internalization and processing for MHC class I-mediated presentation. We can also speculate that the intradermal delivery of the combined Xcl1-Fc + OVA SLP vaccine formulation has reached the inguinal lymph nodes in the form of aggregates, which were engulfed by the same DCs. Additional experiments are required to clarify that aspect. Of note, in our *in vitro* testing, both Xcl1 fusion proteins showed similar binding to Xcr1^+^ DCs as well as similar *in vivo* uptake by CD8α^+^ DCs. Importantly, vaccination with Xcl1 fusion proteins did not only elicit a quantitatively higher CTL response, but also a qualitatively increased recruitment and functionality at the tumor site. In this context, it will be important to evaluate if tumor control could be further enhanced by combining Xcl1-SLP-Fc vaccination with immune checkpoint blockade, as demonstrated by us and others in pre-clinical and clinical settings (26, 34–36). Lastly, it will be also important to study the CD4^+^ T cell response to Xcl1 fusion proteins vaccinations, which we failed to do in this work. Of note, Terhorst et al. (29), who used laser-assisted delivery of Xcl1-OVA fusion protein have reported CD4^+^ T cell responses, which may well-participate in the efficient CD8^+^ T cell priming.

DCs are key players in initiating anti-tumor responses and are considered as an essential target in the context of cancer vaccinations (37). Some cancer vaccines directly target DCs, such as Sipuleucel-T, which is the first FDA-approved DC vaccine for the treatment of refractory prostate cancer (38). Moreover, several clinical trials are currently testing the allogenic GM-CSF-secreting whole tumor cell vaccine GVAX in pancreatic cancer patients (39). However, there is so far no DC vaccine that specifically targets cross-presenting DCs in cancer patients. A harmonization of all the strategies tested so far would help in choosing the best DC-specific receptor(s) for delivering tumor antigens to cross-presenting DCs. Such DC targeting strategies may prove very attractive for personalized cancer vaccines using tumor-derived neo-antigens as identified by mass-spectrometry based antigen discovery (40–42).

Our data demonstrate the applicability of Xcl1/Xcr1-mediated DC vaccine for clinical development, given that Xcr1^+^ cross-presenting DCs have also been well-described in humans. Moreover, developing Xcl1-SLP-Fc fusion proteins as an off-the-shelf DC vaccine might be a more economical and easier alternative to *ex vivo* DC vaccines. Interestingly, the efficacy of the Xcl1-Fc to promote effective targeting of the synthetic long peptide immunogen as a mixture might greatly facilitate the formulation of cancer type-specific, and neo-antigen therapeutic vaccines.

## Author Contributions

NB performed the experiments and participated to the manuscript preparation. BT performed the experiments in the late stage of the study. JH and MS made substantial contributions to conception, experimental design and analysis of results. AD supervised the study and the mansucript preparation. PR designed and supervised the study and manuscript preparation. All co-authors read and approved the final manuscript.

### Conflict of Interest Statement

The authors declare that the research was conducted in the absence of any commercial or financial relationships that could be construed as a potential conflict of interest.
