# Supplementary figures and images for: Combination of Synthetic Long Peptides and XCL1 Fusion Proteins Results in Superior Tumor Control
Source: Front Immunol. 2019 Feb 26;10:294. doi: 10.3389/fimmu.2019.00294 (PMC6399421; doi:10.3389/fimmu.2019.00294)

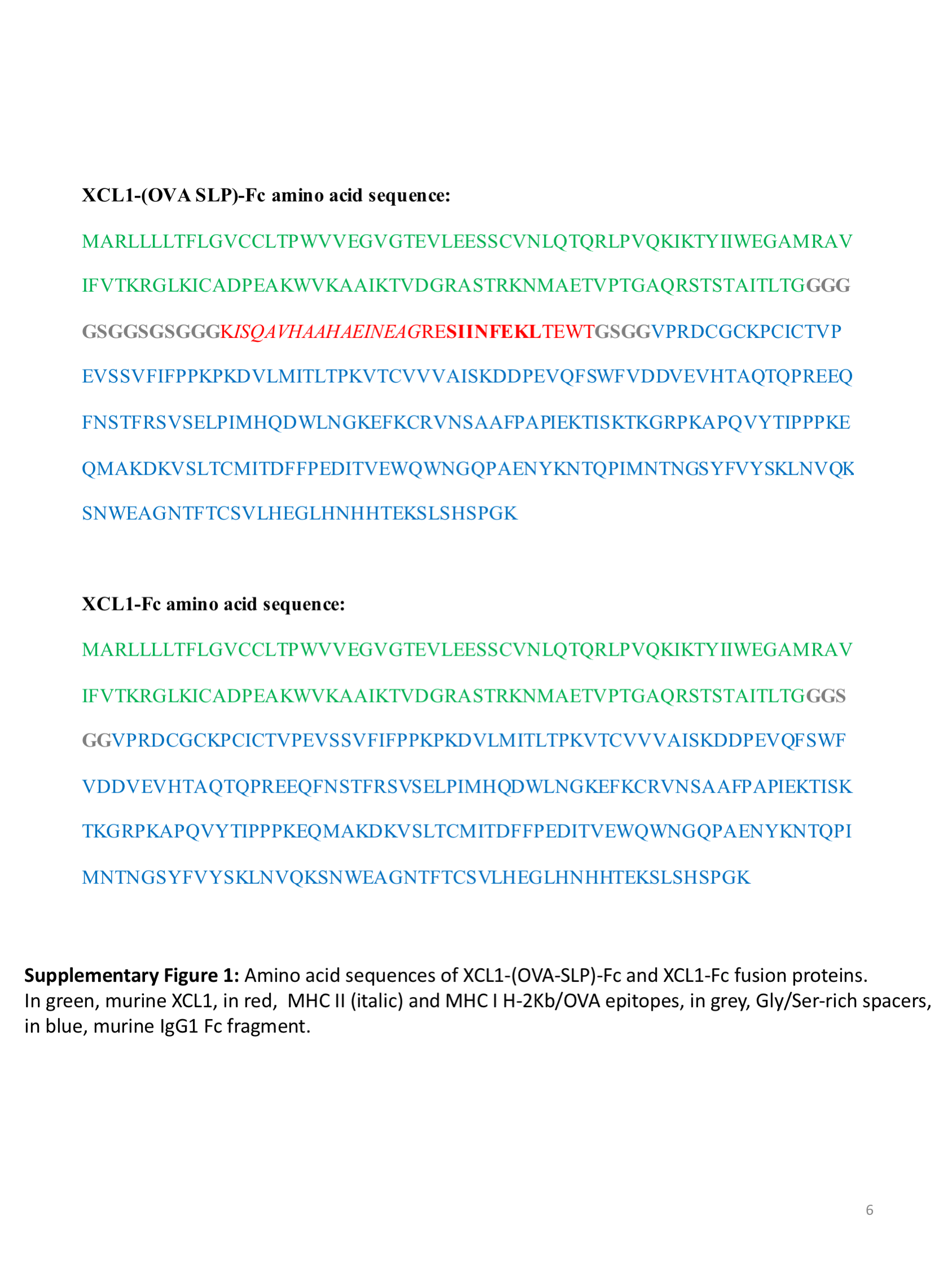

Supplement: Supplementary file 1 [file Image_1.TIFF]

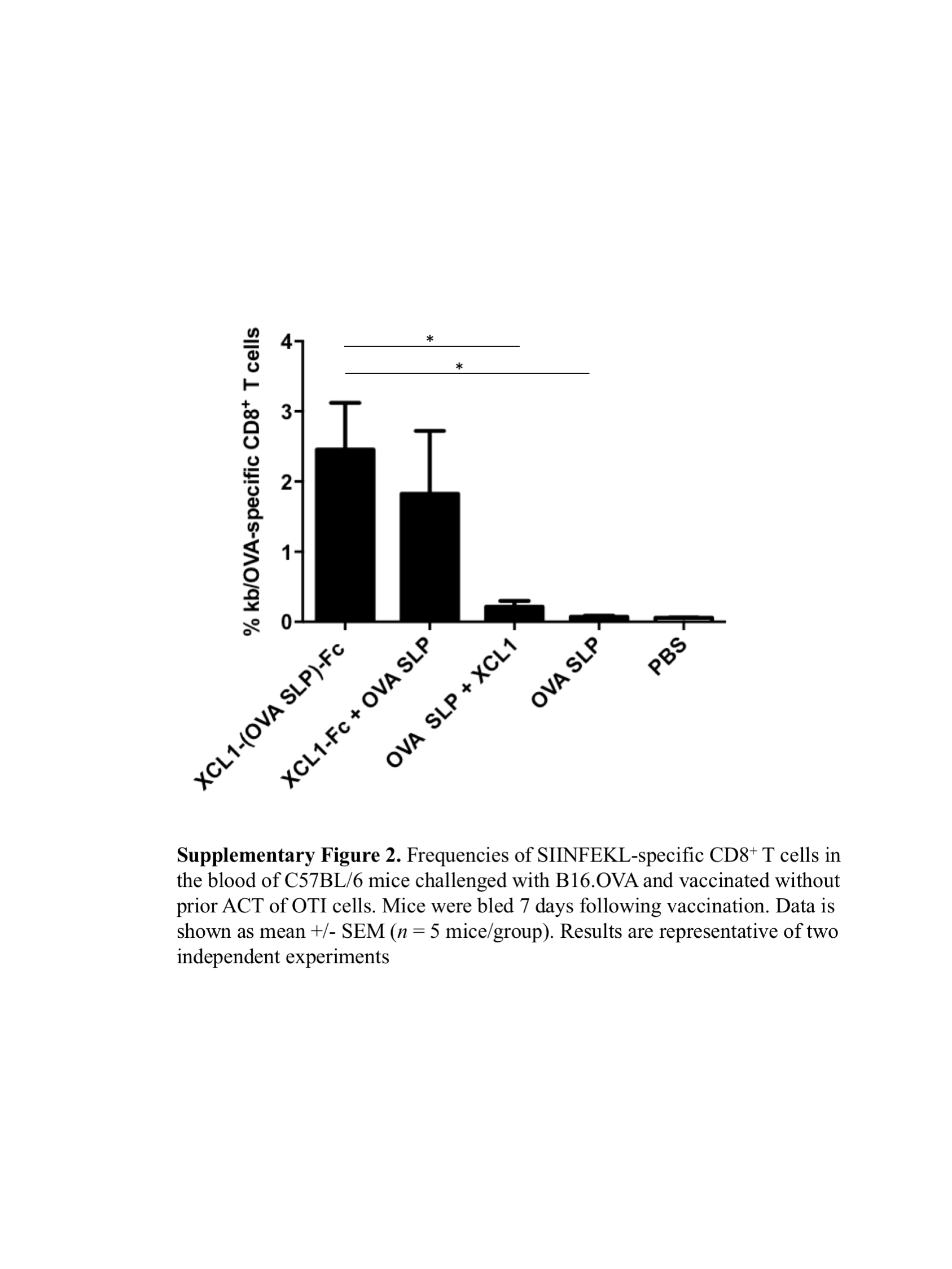

Supplement: Supplementary file 2 [file Image_2.TIFF]

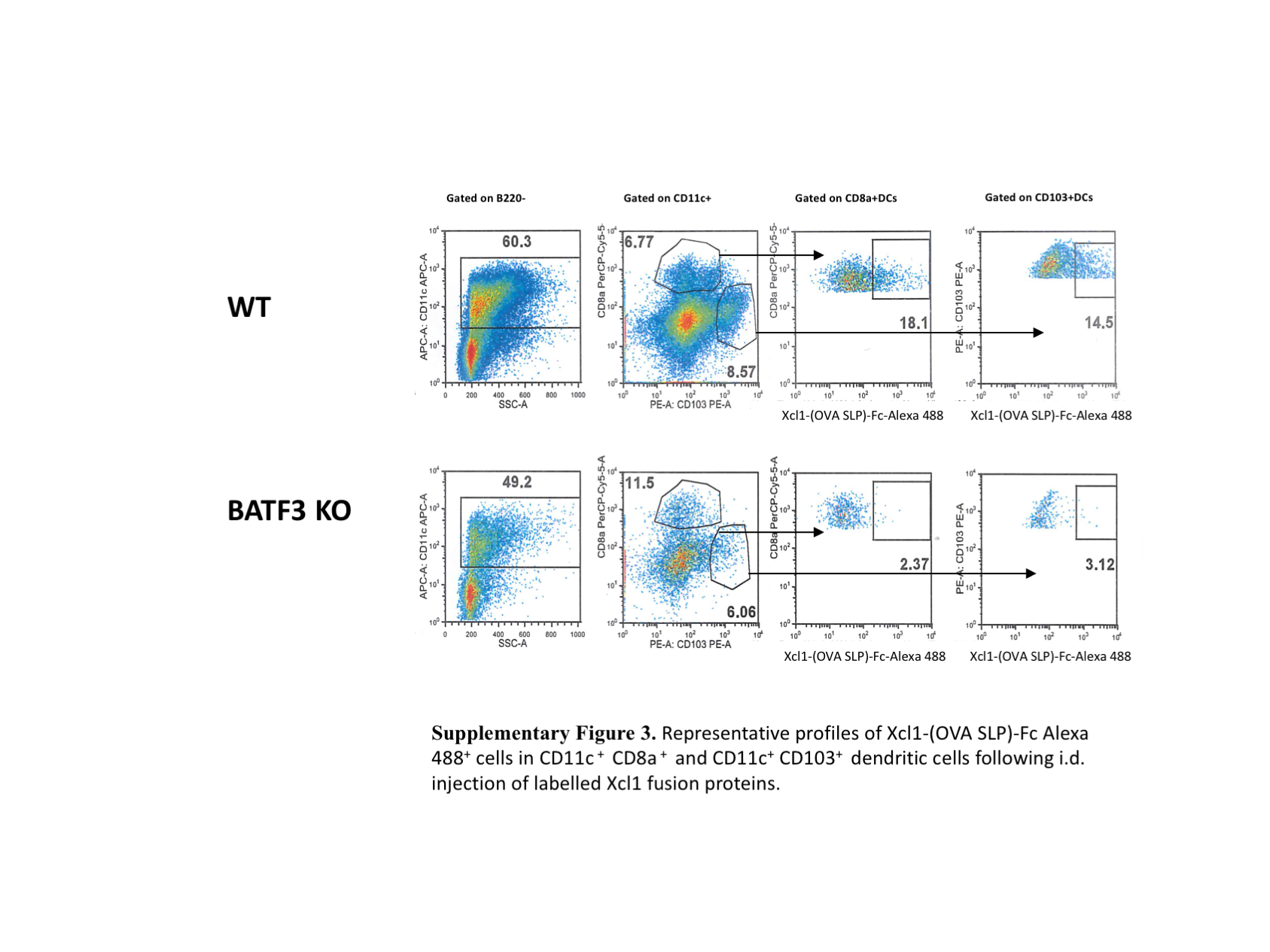

Supplement: Supplementary file 3 [file Image_3.TIFF]

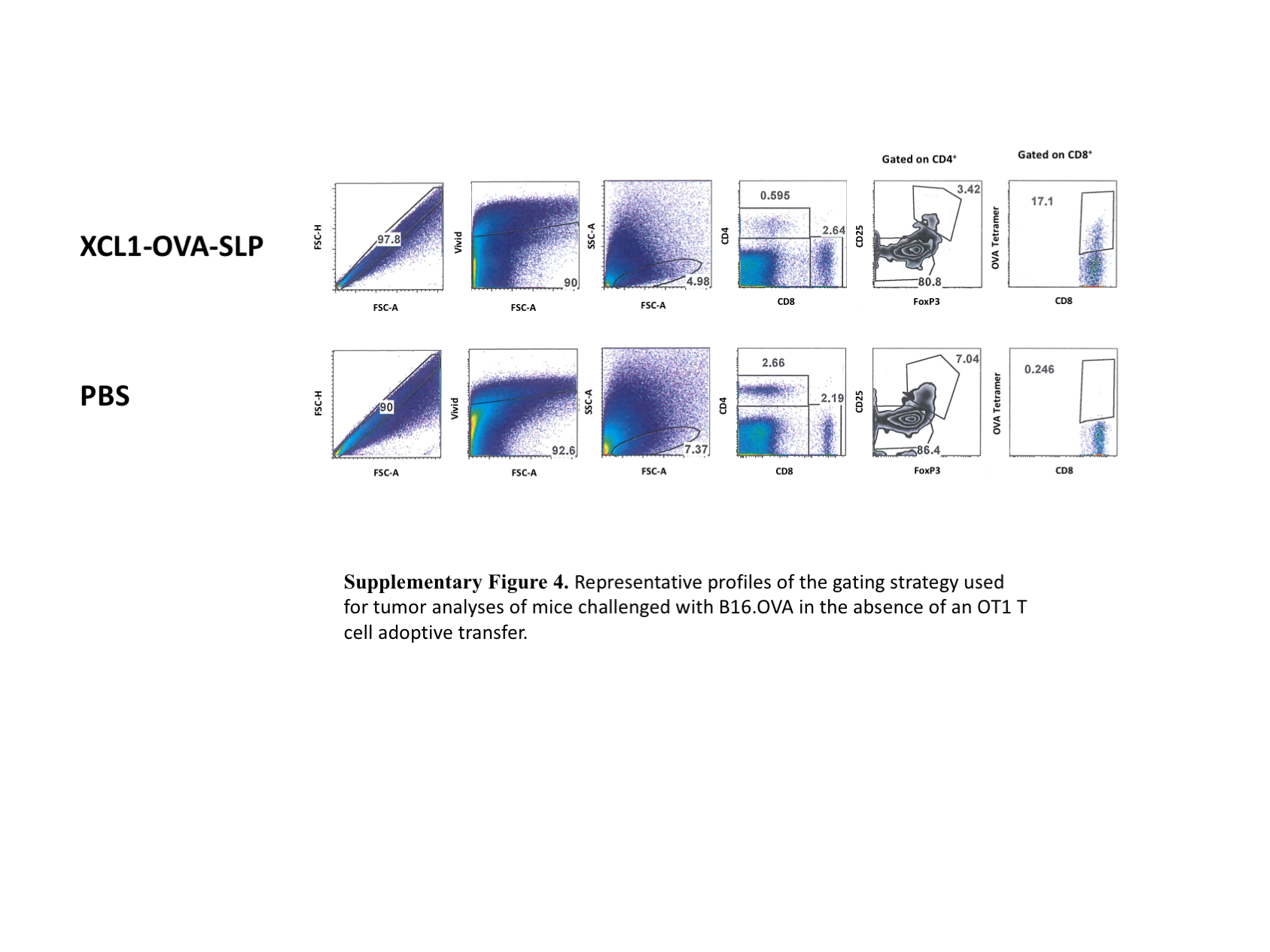

Supplement: Supplementary file 4 [file Image_4.TIFF]
